# Nitrogen additions stimulate litter humification in a subtropical forest, southwestern China

**DOI:** 10.1038/s41598-018-35720-w

**Published:** 2018-12-03

**Authors:** Qun Liu, Liyan Zhuang, Xiangyin Ni, Chengming You, Wanqin Yang, Fuzhong Wu, Bo Tan, Kai Yue, Yang Liu, Li Zhang, Zhenfeng Xu

**Affiliations:** Long-term Research Station of Alpine Forest Ecosystems, Institute of Forest & Ecology, Sichuan Agricultural University, Chengdu, 611130 China

## Abstract

Despite the importance of nitrogen (N) deposition for soil biogeochemical cycle, how N addition affects the accumulation of humic substances in decomposing litter still remains poorly understood. A litterbag experiment was conducted to assess the potential effects of N addition (0 kg·N·ha^−1^·year^−1^, 20 kg·N·ha^−1^·year^−1^ and 40 kg·N·ha^−1^·year^−1^) on mass remaining and humification of two leaf litter (*Michelia wilsonii* and *Camptotheca acuminata*) in a subtropical forest of southwestern China. After one year of decomposition, litter mass was lost by 38.1–46.5% for *M*. *wilsonii* and 61.7–74.5% for *C*. *acuminata*, respectively. Humic substances were declined by 12.1–23.8% in *M*. *wilsonii* and 29.1–35.5% in *C*. *acuminata*, respectively. Nitrogen additions tended to reduce mass loss over the experimental period. Moreover, N additions did not affect the concentrations of humic substances and humic acid in the early stage but often increased them in the late stage. The effect of N addition on the accumulation of humic substances was stronger for *C*. *acuminate* litter than in *M*. *wilsonii* litter. Litter N and P contents showed positive correlations with concentrations of humic substances and fulvic acid. Our results suggest that both litter quality and season-driven environmental changes interactively mediate N impacts on litter humification. Such findings have important implications for carbon sequestration via litter humification in the subtropical forest ecosystems experiencing significant N deposition.

## Introduction

Organic matter accumulated at the surface of forest soils as humus is of primary importance to long-term site fertility and productivity in forest ecosystems^1^. The decomposition and transformation of plant detritus is one of the main processes by which soil organic matter (SOM) is formed^2^. Thus, litter humification plays an important role in improving soil structure, maintaining soil fertility and sequestering soil carbon (C)^3,4^. Litter humification is generally driven by both biotic and abiotic variables, including climate, litter quality, and soil properties^1,5^. In recent years, global N deposition exerts an increasingly crucial role in regulating terrestrial C sink^6,7^. Despite the importance of litter humification for ecosystem fertility and C balance, there remain significant gaps in our understanding as to how N availability impacts this fundamental process.

Global N deposition is anticipated to reach 200 Tg N yr^−1^ in 2050 as a result of fossil fuel combustion and chemical fertilizer use^8^. In China, the rate of N deposition has reached 30–73 kg N ha^−1^ yr^−1^ in tropical and subtropical forests^9^. Several studies have found that N additions produced significant effects on litter decomposition and soil C pools^6,10^. Nitrogen deposition promoted humus accumulation directly by increasing concentration of lignin polymer^4,11^. Additionally, litter humification is driven by matrix quality, such as N concentration, C:N ratio and refractory material^2^. In the N saturated ecosystem, N additions inhibited the degradation of high quality litter (narrow C:N ratio) but increased lignin-like products which are the precursors of humic substances^12–14^. Current studies have focused mainly on litter decomposition rate and nutrient release in N-rich sites^6,14^. However, the dynamic of humic substances in decomposing litter still remains poorly understood.

Subtropical China has a typical rain and heat synchronization climate with rich rainfall and warm temperature^15^. The western edge of Sichuan Bain, known as the rainy zone of western China, is in the center of subtropical zone. The annual wet N deposition in this study site is 36.2 kg N·ha^−1^ ^16^, which is much higher than the average value of atmospheric N deposition in China (18.0 kg N ha^−1^)^17^. Thus, this region is considered as a special natural laboratory for N deposition studies of N-rich ecosystem. Previous studies have mainly focused on the mass loss and elements releases of plant litter, soil respiration and C pools under N addition conditions^6,18,19^. To better understand the effects of rising N deposition on soil organic matter, a field experiment was conducted to explore the potential influences of N additions on the humification of two foliar litter with contrasting quality (*Michelia wilsonii* and *Camptotheca acuminata*) in the high-N deposition subtropical ecosystem. Specifically, we hypothesize that (1) N additions would stimulate litter humification and such effect could be greater in high-quality litter; (2) season-associated conditions could mediate the response of litter humification to N additions.

## Results

### Mass remaining

**Figure 1:**
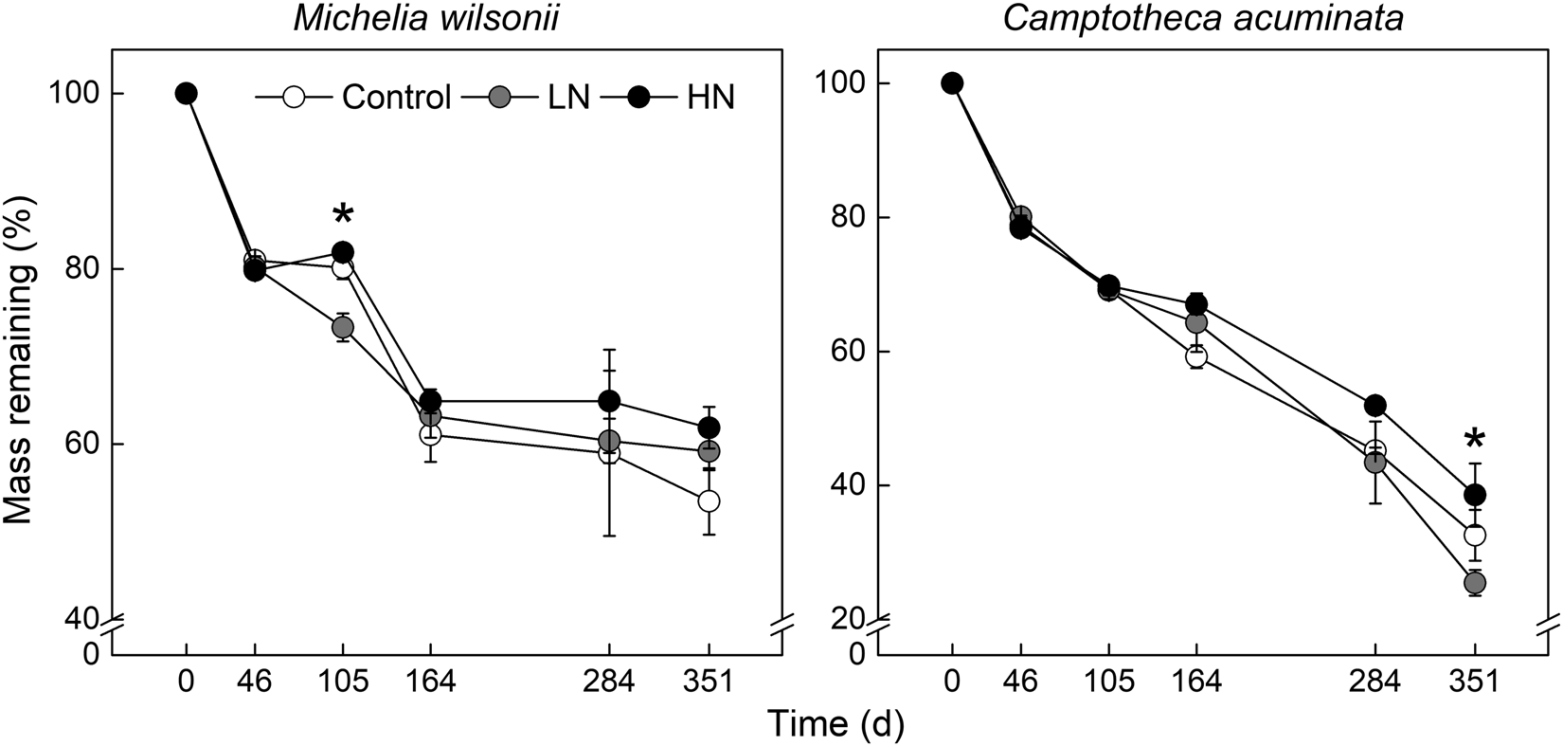
Mass remaining of *M. wilsonii* and *C. acuminata* over the experimental period under three N treatments. Control; LN, low-N treatment; HN, high-N treatment. *P < 0.05.

N addition treatments had marginal effects on litter mass remaining (P = 0.08, Table 1). In general, N additions tended to increase litter mass remaining over the experimental period (Fig. 1). ANOVA analysis showed that the effect of N treatment on mass remaining was dependent on deposition period (Table 1). Regardless of N treatments, *C. acuminata* litter often decomposed faster than did *M. wilsonii* litter (Fig. 1). After one year of decomposition, litter mass was lost by 38.1–46.5% for *M. wilsonii* and 61.7–74.5% for *C. acuminata*, respectively (Fig. 1).

**Table 1:** Results of repeated measures ANOVA testing for the effects of nitrogen addition (N), litter type (L), sampling date (S) and their interactions on litter mass remaining and humic substances, humic acid, fulvic acid and humic acid/fulvic acid.

Factor	Mass remaining		Humic substances		Humic acid		Fulvic acid		Humic acid/fulvic acid	
	*F*	*P*	*F*	*P*	*F*	*P*	*F*	*P*	*F*	*P*
N	1.60	0.08	7.785	0.007	6.134	0.015	15.238	0.001	7.785	0.069
L	21.65	<0.001	48.335	<0.001	156.831	<0.001	6.426	0.026	48.335	<0.001
S	120.33	<0.001	69.731	<0.001	294.645	<0.001	81.537	<0.001	69.731	<0.001
N × S	2.84	0.031	5.928	<0.001	15.299	<0.001	4.356	0.001	5.928	<0.001
L × S	19.59	<0.001	14.532	<0.001	39.932	<0.001	14.537	<0.001	14.532	<0.001
N × L	0.03	0.945	0.108	0.898	0.109	0.898	4.822	0.029	0.108	0.107
N × L × S	1.23	0.338	1.974	0.127	8.189	<0.001	4.869	<0.001	1.974	<0.001

### Humic substances

**Figure 2:**
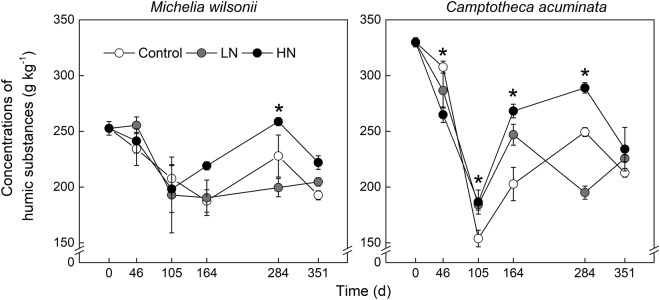
Humic sustances concentration of *M*. *wilsonii* and *C*. *acuminata* over the experimental period under three N treatments. Control; LN, low-N treatment; HN, high-N treatment. *P < 0.05.

Concentrations of humic substances remarkably varied along the decomposition advancement (Table 1, Fig. 2). N additions did not affect humic substances for both litter types at the early stage of decomposition. However, N treatments, especially HN treatment, often stimulated humic substances over the late decay period (Fig. 2). In addition, N additions appeared to induce greater impacts on *C. acuminata* as compared to *M. wilsonii*. After one-year field incubation, concentration of humic substances in *M. wilsonii* was reduced by 23.8%, 19.0% and 12.1% for Control, LN and HN treatments, respectively. Likewise, concentration of humic substances in *C. acuminata* was decreased by 35.5%, 31.6% and 29.1% for Control, LN and HN treatments, respectively (Fig. 2). Statistical analysis indicated that the interactions of both N treatment × time and species × time had significant effects on humic substances (Table 1).

### Humic acid

**Figure 3:**
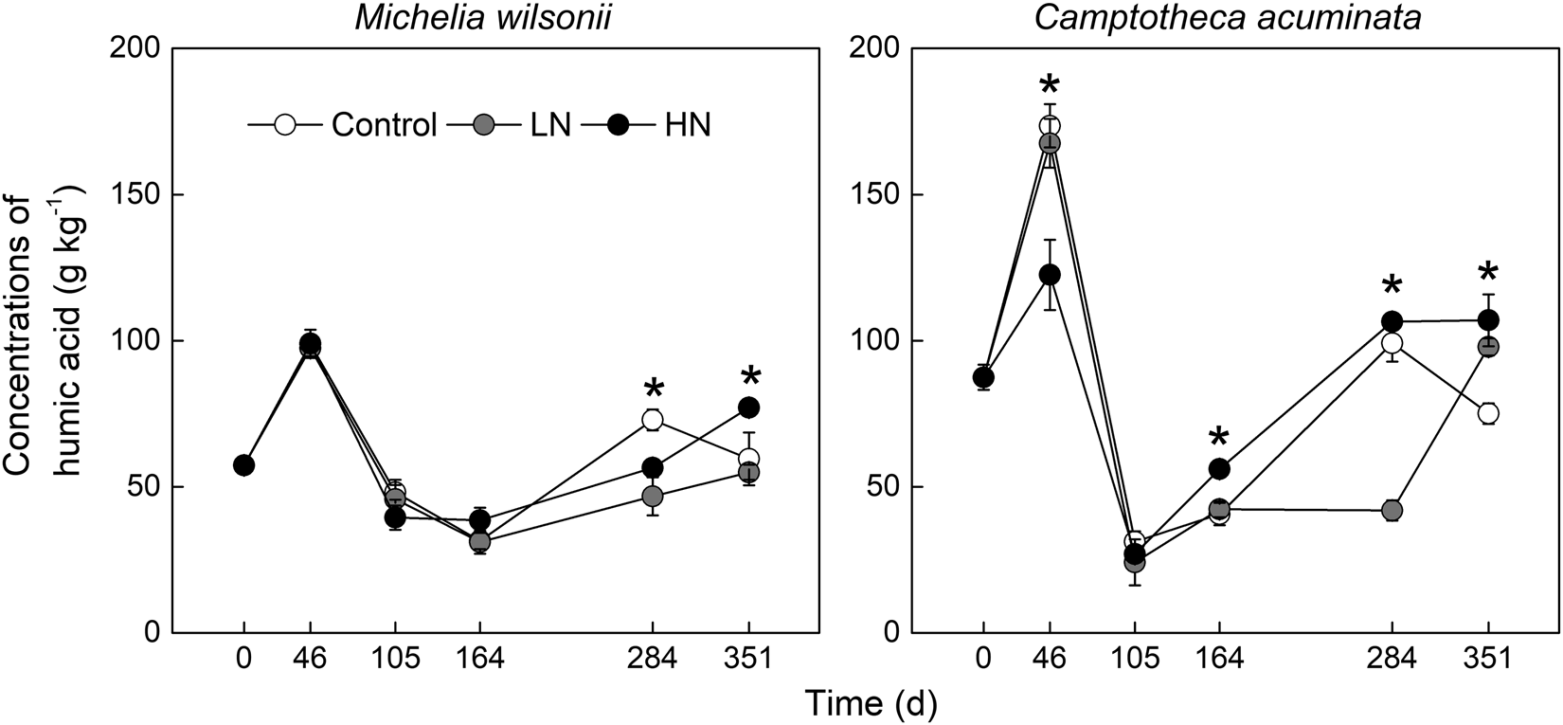
Humic acid concentration of *M*. *wilsonii* and *C*. *acuminata* over the experimental period under three N treatments. Control; LN, low-N treatment; HN, high-N treatment. *P < 0.05.

Concentrations of humic acid significantly varied with sampling date (Table 1, Fig. 3). Similarly, N treatments did not affect the concentration of humic acid on both litter types at the early period. However, N treatments, especially HN treatment, tended to decline humic acid during the late period (Fig. 3). By the end of the experiment, humic acid was declined by 3.88–34.34% in *M. wilsonii* and by 11.95–22.35% in *C. acuminata*, respectively (Fig. 3). ANOVA analysis indicated that the effect of N treatments on humic acid was dependent on litter types and decomposition period (Table 1).

### Fulvic acid

**Figure 4:**
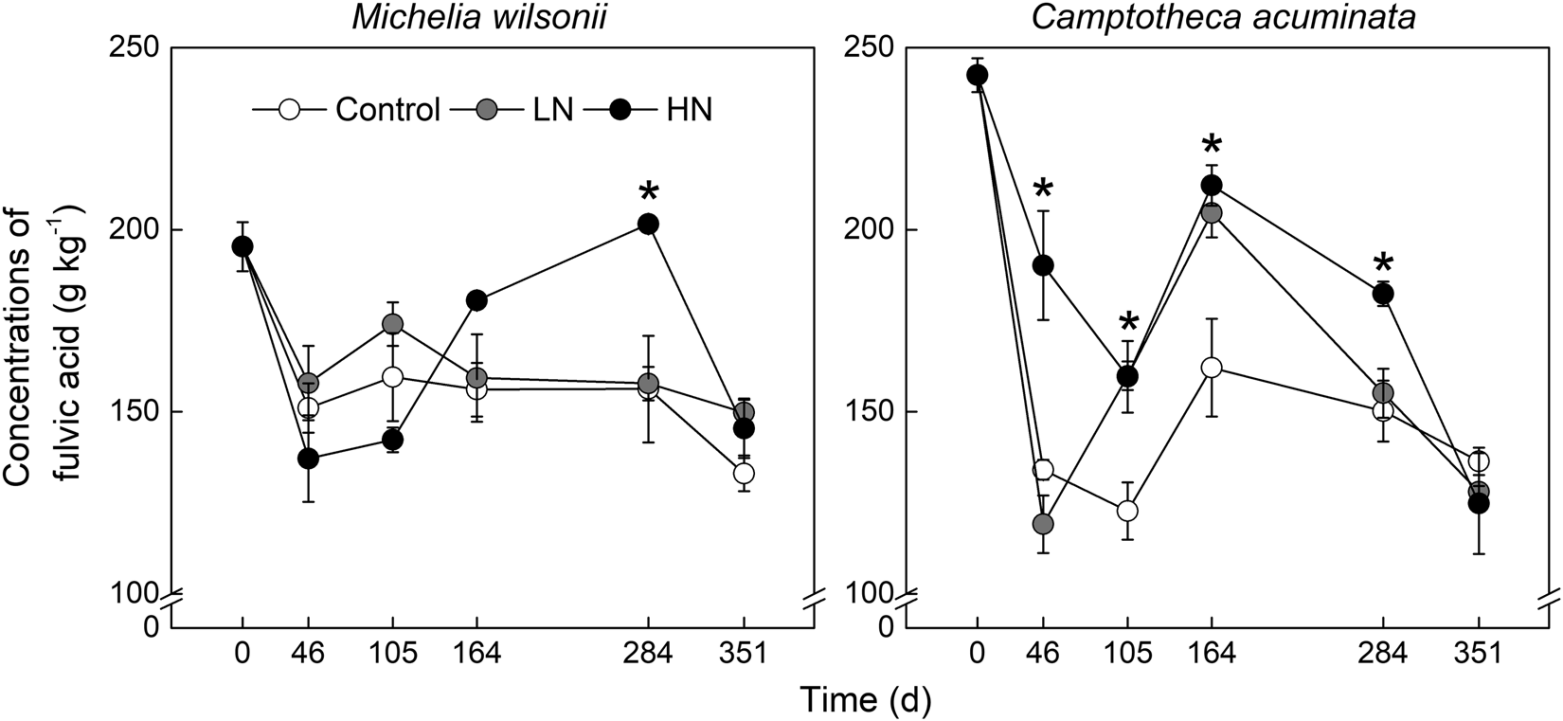
Fulvic acids concentration of *M*. *wilsonii* and *C*. *acuminata* over the experimental period under three N treatments. Control; LN, low-N treatment; HN, high-N treatment. *P < 0.05.

Similar to humic acid, N treatments did not affect and even reduced the concentration of fulvic acid in both litter types in the early decomposition period. Conversely, N treatments often stimulated fulvic acid during the later decay period (Fig. 4). In addition, N additions appeared to induce greater impacts on *C. acuminata* as compared to *M. wilsonii*. After 1-year field incubation, fulvic acid was reduced by 31.88%, 23.36% and 25.56% for the Control, LN and HN treatments in *M. wilsonii*, respectively; and by 43.75%, 47.21% and 48.51%, respectively, in *C. acuminata* (Fig. 4). Statistical analysis showed that the interactions of both N treatment × time and species × time had significant effects on fulvic acid (Table 1; P < 0.001).

### Humic acid/Fulvic acid

**Figure 5:**
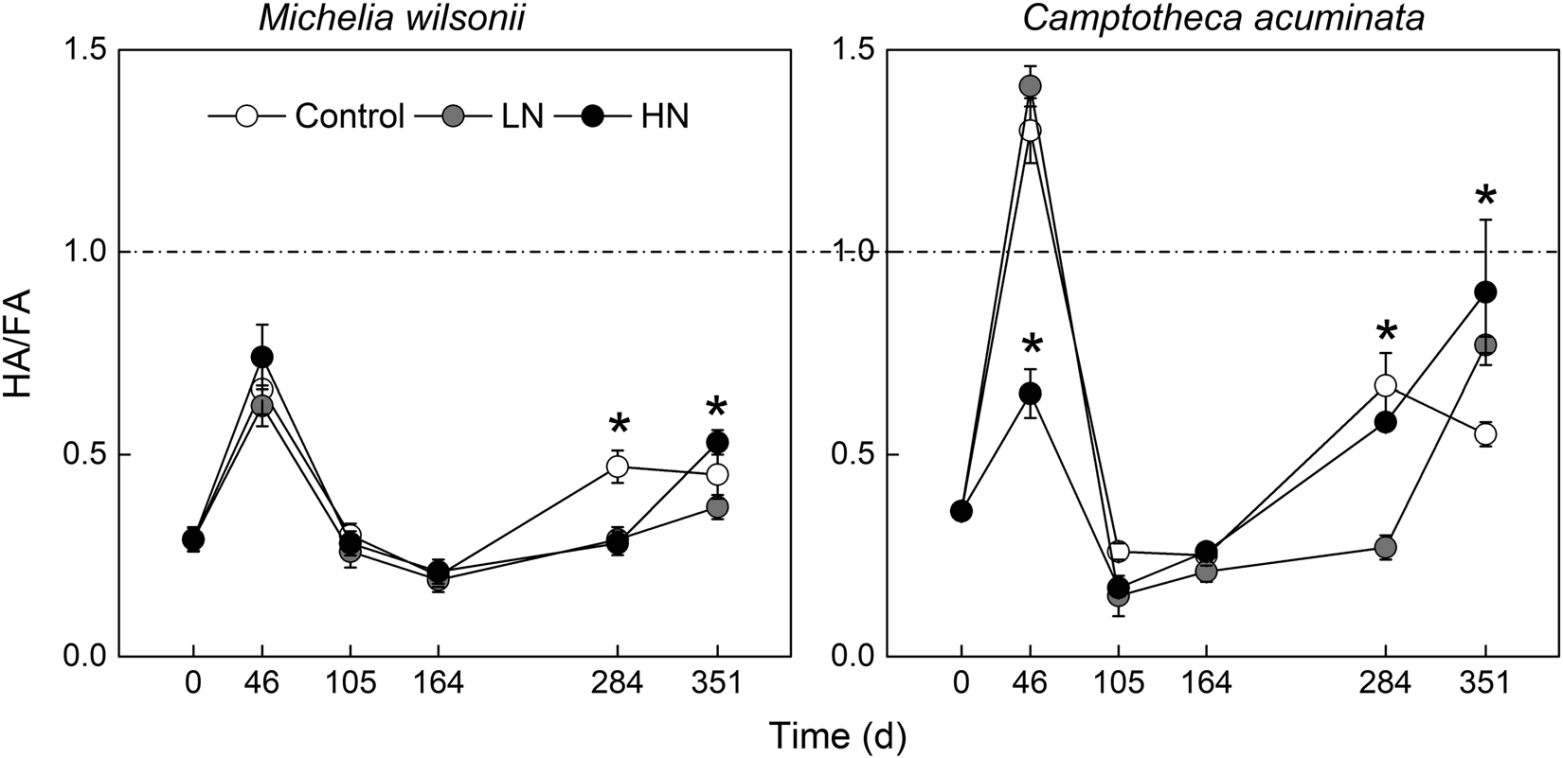
HA/FA value of *M*. *wilsonii* and *C*. *acuminata* over the experimental period under three N treatments. Control; LN, low-N treatment; HN, high-N treatment. *P < 0.05.

The HA/FA value largely varied with decomposition period (Table 1, Fig. 5). N treatments did not affect HA/FA for both litter types in the early decomposition period. However, N treatments, especially LN treatment, tended to decrease HA/FA values on the 284 and 351 days of decomposition (Fig. 5). Additionally, N additions induced greater impacts on *C. acuminata* relative to *M. wilsonii*. Apart from the first sampling stage (46 days) for *C. acuminata*, the HA/FA value of two species was always less than 1 throughout the experimental period (Fig. 5). Statistical analysis indicated that the effect of N treatments on HA/FA was significantly dependent on deposition stages and litter types (Table 1).

### Correlations between nutrients and humic matter

In general, there were significant positive correlations between N and P contents and concentrations of humic substances and humic acid. The ratio of N:P showed a negative relationship with concentration of humic substances and humic acid. However, there were no significant correlations between concentration of fulvic acid and N, P and N:P (Table 2).

**Table 2:** Results of correlation analysis among nutrient concentrations and the concentrations of humic substances, humic acid and fulvic acid at each sampling period. (n = 18).

Factor	First sampling			Second sampling			Third sampling			Fourth sampling			Fifth sampling		
	HS	HA	FA	HS	HA	FA	HS	HA	FA	HS	HA	FA	HS	HA	FA
N	0.719**	0.808**	0.006	−0.427	−0.691**	−0.182	0.667**	0.618**	0.650**	0.464	0.508*	0.139	0.227	0.537*	−0.207
P	0.684**	0.834**	−0.22	−0.428	−0.735**	−0.249	0.460	0.613**	0.380	0.560*	0.594**	0.207	0.723**	0.743**	−0.485*
N:P	−0.642**	−0.830**	0.037	0.065	0.183	0.089	−0.086	−0.367	0.021	−0.497*	−0.388	0.279	−0.639**	−0.586*	0.200

*P < 0.05; **P < 0.01.

## Discussion

Litter decomposition is a fundamental biogeochemical process that is remarkably influenced by environmental factors^20,21^. N deposition has been increasingly recognized as one of important global change factors that regulates litter decomposition rate of terrestrial ecosystems^6,22^. Over last decades, N deposition effects on litter decomposition have extensively been reported in diverse terrestrial ecosystems^23,24^. However, inconsistent patterns have been observed. For instance, decreases in litter mass loss in response to N additions were reported in tropical and subtropical forests^6,22,25^. In some grassland ecosystems, experimental N depositions showed no effect on litter decay rates^26,27^. However, N additions promoted litter decay in a lowland Panamanian forest^28^ and in a temperate forest in Patagonia^29^. In this study, N addition treatments exhibited a slight negative impact on litter mass loss of two tree species. On the other hand, a meta-analysis showed that the effects of N additions on plant litter decomposition were dependent strongly on fertilization rates and litter quality^23^. Specifically, high N addition rates tended to inhibit the decomposition of high-lignin litter but low N addition rates often stimulated the decomposition with low-lignin litter^23^.

Litter humification is a complex biogeochemical process for soil organic matter accumulation that is strongly influenced by biotic and abiotic factors^1,3^. Previous studies have shown that litter humification largely varied among plant species^4,30^. In this case, irrespectively of N treatments, the humic substances of *C*. *acuminata* litter accumulated faster than did *M*. *wilsonii* litter. Litter humification is strongly driven by litter quality, such as N concentration, C:N ratio and refractory materials^2,4,30^. Our study also found that N and P contents were often correlated positively with humic substances and humic acid. *C*. *acuminata* litter (a deciduous broad-leaved tree species) had higher N and P contents relative to *M*. *wilsonii* litter (an evergreen broad-leaved tree species), implying that the humification rate of *C*. *acuminata* litter was faster as compared to that of *M*. *wilsonii* litter.

A growing number of studies have indicated that rising atmospheric N deposition resulted in significant impacts on soil organic matter accumulation^1,31^. In this study, N additions were favorable to litter humification of two subtropical forest tree species. Similar results were reported in temperate and boreal forests^32,33^. The positive effects of N additions on litter humification could be attributed to biological inhibitions of lignin-degrading enzymes and chemical stimulations of humus formation^34–36^.

In addition, the humification of *C*. *acuminata* litter was more responsive to N addition as compared to *M*. *wilsonii* litter. The differences in matrix litter quality could partly account for the observed responses between the two litters. The labile components of *C*. *acuminata* litter (deciduous broad leaved tree species) are often higher than those of *M*. *wilsonii* litter (evergreens broad leaved tree species). Labile components are the dominant source of microbial products because they are utilized more efficiently by microbes^2^. Such products could thus become the main precursors of humus by condensation reactions and strong chemical bonding^2,36^. On the other hand, exogenous N is able to bind with substrate lignin to form humus^36^. N additions also inhibited lignin degradation via depressing the activity of lignin-degrading enzyme^35^. High-quality litter with narrow C: N were more sensitive to N additions^13,14^. As a result, N additions caused greater effects on the humic acid and fulvic acid of *C*. *acuminata* as compared to those of *M*. *wilsonii*. Thus, litter quality itself may, to large extent, mediate the humification magnitude of decomposing litter to atmospheric N deposition, while further evidence is needed for confirmation.

In addition, the effect of N deposition on litter humification could also vary along the decomposition advancement. Previous studies have found that N additions had different impacts on litter humification in different decomposition stages^11,36^. In our study, N additions did not affect the concentrations of humic substances, humic acid and fulvic acid of the two leaves litter at the early stage. However, N additions, especially high N treatment, often stimulated humification over the late decay period. In the early experimental period, microbes need to immobilize N from litter and soil to meet the requirements for growth and maintenance^37^. As a consequence, N additions can not have significant impacts on litter humification in this period^38,39^. However, over the late stage, when litter decomposition is dominated by lignin and lignin-like products, exogenous N may react with the aromatic compounds within litter to form humic substances^4,40^. In addition, high N treatment decreased humic acid but increased fulvic acid of *C*. *acuminata* at the initial stage, implying that fulvic acid may be transformed to humic acid at this stage.

In general, the composition of humus is characterized by HA/FA, which reflects the relative rates of formation of humic acid and fulvic acid^41,42^. Some studies indicated that fulvic acid can be formed first during the humification process when HA/FA is lower than 1^41^. In this study, the HA/FA ratios of two tree species were always smaller than 1, suggesting that the nonstructural carbohydrates in both subtropical tree leaf litter are preferable to be converted to fulvic acid rather than humic acid during the decomposition process. Similar observations have been found in leaf and root litters of subalpine tree species^30,43^. In addition, the newly formed humic acid with unstable structure and low condensation degree of aromatic nucleus is easily transformed into fulvic acid^44^. The relatively high HA/FA ratios of *M*. *wilsonii* on the first 46^th^ days of decomposition is mainly attributed to both significant degradation of fulvic acid and accumulation of humic acid. In general, N additions did not affect the HA/FA ratios of two litter types during the early decomposition period, suggesting that degradation and accumulation of two components is similar among N treatments. However, N additions tended to increase HA/FA ratios for two tree species by the end of incubation experiment, implying that N additions may favor transformation from fulvic acid to humic acid. Such conversion could, to some extent, make humus structure within the litters more stable^5^.

## Conclusions

In general, N treatments, especially high N addition, tended to inhibit mass loss of two foliar litter over one-year experimental period. However, N additions often increased humic substances, humic acid and fulvic acid especially in the late decomposition period, suggesting that N additions are favorable to the humification in decomposing litters. However, the effect size of N additions was different between two contrasting species. The humification of the deciduous high-quality *C*. *acuminata* litter was stronger than that of the evergreen low-quality *M*. *wilsonii* litter. Regardless of N treatments and tree species, measured variables involved in humification exhibited similar seasonal patterns. Moreover, season-caused changes were much stronger than N-induced variations or interspecific differences. Therefore, season-driven environmental changes may be the main determinant of litter humification in this region.

## Materials and Methods

### Study site

The study is conducted in the Dujiangyan Experimental Forest of Sichuan Agricultural University, southwestern China (31°01′-31°04′N, 103°37′-103°43′E, 896–1320 m a.s.l.). The annual mean temperature is 15.2 °C, with the lowest temperature being −1.4 °C in January and the highest temperature being 31.6 °C in July. The annual mean precipitation is approximately 1243 mm. The ambient wet N deposition is 36.2 kg N ha^*−*1^ yr^*−*1^ ^16^. A mixed forest of *M. wilsonii* and *C. acuminata* was selected in this study. The soil is classified as ferralsol with old alluvial yellow loam according to the Chinese Soil Taxonomy (RGCST 2001). The concentration of C, N and P in the 0–20 cm soils were 15.76 g kg^−1^, N 1.92 g kg^−1^, P 0.32 g kg^−1^ respectively, and the soil pH was 5.73.

### Experimental design

In November 2015, nine 10 × 10 m plots (3 replicate plots per treatment) with 10 m intervals were established. According to the ambient atmospheric wet N deposition (36.2 kg N·ha^−1^) in this study area^16^, three N addition levels were set up for this experiment: control (Control: without N addition), low N addition (LN: 20 kg N ha^−1^ yr^−1^) and high N addition (HN: 40 kg N ha^−1^ yr^−1^). Both LN and HN treatments simulated the scenarios that wet N deposition is increased by about 50% and 100%, respectively. NH_4_NO_3_ solution was sprayed monthly from December 2015 to December 2016. Briefly, 47.6 g and 95.2 g NH_4_NO_3_ were weighed and mixed with 10 L water for LN and HN treatments, respectively. The NH_4_NO_3_ solution was evenly sprayed on the surface soil and litter bags. The control plots received equivalent 10 L water without NH_4_NO_3_.

**Figure 6:**
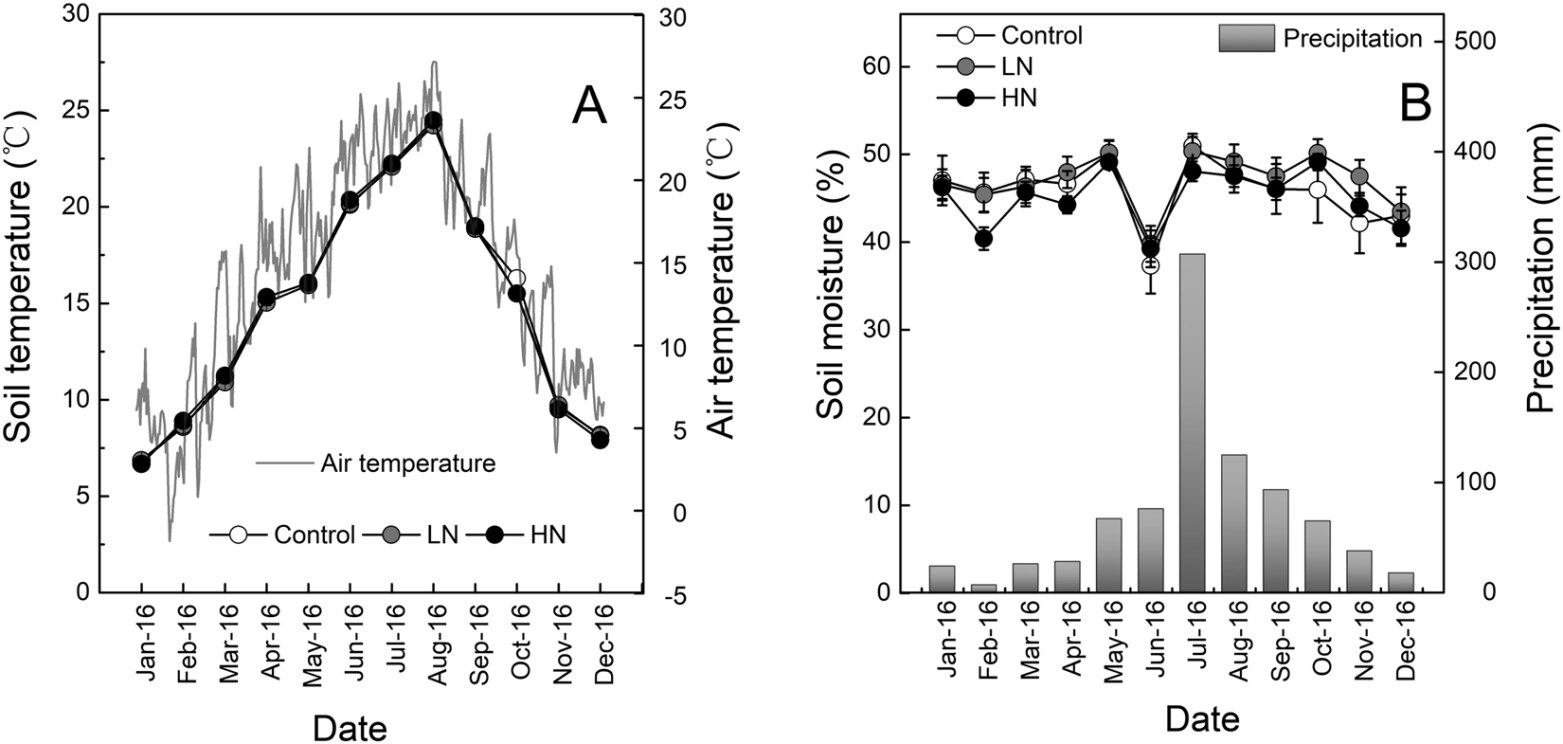
Seasonal dynamics of air and soil temperatures (**A**) and soil moisture and monthly precipitation (**B**) during the experimental period. Control; LN, low-N treatment; HN, high-N treatment. *P < 0.05.

In late October 2015, fresh leaf litter of two tree species differing in initial chemistry were sampled from this forest stand (Table 3). Air-dried litters (15.00 ± 0.01 g for each tree species) were placed into 20 × 25 cm nylon mesh bags with a 1.0-mm mesh on the surface and 0.055-mm mesh on the bottom. A total of 216 litter bags (3 N addition levels × 2 species × 6 sampling dates × 6 replicates) were deployed on the soil surface in the respective treatment section in the early January 2016. Meanwhile, two litter bags of each species were retrieved from each plot for determination of initial chemical properties. Two litter bags of each species were randomly harvested from each plot 46, 105, 164, 284 and 351 days after field incubation. In the laboratory, extraneous materials such as plant materials, rocks and soil particles were removed from the decomposed litter, and the clean samples were then oven-dried at 85 °C to a constant mass. In addition, air temperature (2 m height) was measured using DS1923-F5 Recorders (iButton DS1923-F5, Maxim/Dallas Semiconductor, Sunnyvale, USA) and soil temperature at the depth of 5 cm was measured by a thermocouple probe (Fig. 6A). Monthly precipitation data was collected from the local weather bureau and soil moisture at the depth of 5 cm was determined by a Theta probe (Fig. 6B).

**Table 3:** Initial chemistry of *M*. *wilsonii* and *C*. *acuminata* leaf litters.

Species	C (g kg^−1^)	N (g kg^−1^)	P (g kg^−1^)	Lignin (%)	Cellulose (%)	C/N	C/P	N/P	Lignin/N
*M*. *wilsonii*	559.4 ± 6.2^a^	10.3 ± 0.3^a^	0.5 ± 0.05^a^	19.4 ± 0.7^a^	14.9 ± 4.1^a^	54 ± 1^a^	1192 ± 126^a^	22 ± 2^a^	1.9 ± 0.06^a^
*C*. *acuminata*	558.3 ± 7.8^a^	15.7 ± 1.2^b^	1.0 ± 0.12^b^	10.3 ± 0.5^b^	8.4 ± 1.7^b^	36 ± 3^b^	560 ± 58^b^	16 ± 2^b^	0.7 ± 0.07^b^

Different lowercase letters indicate a significant difference between two tree species.

### Chemical analysis

Humic substances were extracted with 100 ml mixed solution of 0.1 ML^−1^ NaOH + 0.1 ML^−1^ Na_4_P_2_O_7_ using 1.00 g air-dried foliar litter^45^. Humic acid and fulvic acid were separated with 0.5 ML^−1^ H_2_SO_4_ at 80 °C, and the separated humic acid was dissolved with hot 0.05 ML^−1^ NaOH solution. Both humic substances and humic acid were passed through a 0.45 μm filter and then analyzed using a TOC analyzer (multi N/C 2100, Analytik Jena, Thüringen, Germany).

### Calculations and statistical analysis

Mass remaining and concentration of fulvic acid (FA)^30^ were calculated as follows:

$$\begin{array}{c}{\rm{Mass}}\,{\rm{remaining}}( \% )={M}_{t}/{M}_{0}\times 100 \% \\ FA(g\,k{g}^{-1})=HS-HA\end{array}$$

where M_0_ and M_t_ are the oven-dried mass of initial and the oven-dried remaining masses at times t; *HS* and *HA* are the concentrations of humic substances and humic acid; the relative concentration and formation rate of humic acid and fulvic acid were characterized by humic acid to fulvic acid ratio (HA/FA) on each sampling date^41^.

Significant differences in initial chemistry between two tree species were determined using student *t*-test. A repeated measure analysis of variance (ANOVA) was used to test the effects of tree species, N addition treatments, sampling data and their interactions on measured variables. At each sampling time, the differences among N addition treatments were evaluated using one-way ANOVA with Fisher’s LSD test. A correlation analysis was used to test the relationships between nutrient concentrations and the concentrations of humic substances, humic acid and fulvic acid at each sampling period. The statistical tests were considered significant at the *P* < 0.05 level. All statistical tests were performed using the Software Statistical Package for the Social Sciences (SPSS) version 17.0 (IBM SPSS Statistics Inc., Chicago, IL, USA).
